# Association between peroxisome proliferator-activated receptor γ-2 gene Pro12Ala polymorphisms and risk of hypertension: an updated meta-analysis

**DOI:** 10.1042/BSR20190022

**Published:** 2019-02-27

**Authors:** Miao Zhang, Jianping Zhang, Lifeng Li, Qiang Wang, Limin Feng

**Affiliations:** 1 Tianjin University of Traditional Chinese Medicine, Tianjin City 301617, China; 2 Department of Cardiology, The Second Affiliated Hospital of Tianjin University of Traditional Chinese Medicine, Tianjin City 300250, China; 3 Department of Traditional Chinese Medicine, Tianjin Public Security Hospital, Tianjin City 300042, China; 4 Department of VIP Ward, The Second Affiliated Hospital of Tianjin University of Traditional Chinese Medicine, Tianjin City 300250, China

**Keywords:** Hypertension, meta-analysis, polymorphism, peroxisome proliferator-activated receptor

## Abstract

Previous studies investigate the relationship between peroxisome proliferator-activated receptor γ-2 (*PPAR*) gene Pro12Ala polymorphisms and risk of hypertension. However, the number of available studies was extremely limited. We updated this evidence and gave more significant results. We performed comprehensive computer-based searches in the PubMed, Web of Science, Embase, Google Scholar, the Cochrane library, Wanfang database, China National Knowledge Infrastructure, and China Biological Medicine Database. All studies that reported the association between the PPARγ2Pro12Ala polymorphisms and hypertension were identified. Twenty-one studies were finally included in the present study. In the domain model, the PPARγ1Pro12Ala polymorphism was not associated with hypertension (odds ratio (OR) = 0.85, 95% confidence interval (CI): 0.71–1.03, *P*=0.108). The significant relationship was found in the recessive model (OR = 0.67, 95% CI: 0.53–0.85), in the additive model (OR = 0.61, 95% CI: 0.48–0.77), and in the allele genetic model (OR = 0.81, 95% CI: 0.66–0.99). Subgroup analysis indicated that the PPARγ1Pro12Ala polymorphism from the all gene models was also not related to the risk of hypertension in Caucasians. In Asians, however, the results (*P*=0.002; *I^2^* = 57.6%) suggested a significant relationship between PPARγ1Pro12Ala and hypertension in the domain model (OR = 0.80, 95% CI: 0.65–0.99), in the recessive model (OR = 0.57, 95% CI: 0.44–0.75), in the additive model (OR = 0.51, 95% CI: 0.39–0.66), and in the allele model (OR = 0.75, 95% CI: 0.60–0.94). The PPARγ1Pro12Ala polymorphism could affect the risk of primary hypertension amongst Asians. The A allele gene was a protective genotype for primary hypertension. The PPARγ1Pro12Ala polymorphism was not associated with hypertension amongst Caucasians.

## Introduction

Cardiovascular disease is the leading cause of death for adults in developed or developing countries, including China [1]. Hypertension has been proved the primary and independent modifiable risk factor for stroke and coronary heart disease so that controlling hypertension is one of the most effective ways to prevent cardiovascular disease [2]. Hypertension amongst adults with no identifiable cause is essential hypertension. Essential hypertension as complex quantitative trait is affected by varying combinations of genetic and environmental factors. Probably, genetic factor influences the blood pressure through numerous minor-effect genes, and biological process of hypertension involves multiple physiological pathways with gene and environmental factors [3]. Recently, two-stage association study-based community population is a cost-effective approach for identifying complex disease genes in genetic studies and it has received much attention. Researchers have conducted lots of association studies and identified some potential candidate genes in hypertension.

Peroxisome proliferator-activated receptor-γ (PPARγ) belongs to member of the steroid hormone receptor superfamily. Human PPARγ gene is located in the chromosome of 3p25 with 479 amino acid resides [4]. The gene’s full length is 146485 bp. PPAR gene is the one of the PPARs subtypes that is by far the most widely studied. It is not only the target molecular of Thiazolane diketones but also an important regulatory factor of adipocyte differentiation and fat endocrine functions of the important regulatory factor [5]. Moreover, PPAR gene acted as a signal transfer regulator between gene expression of adipocytes and adipogenesis and insulin-producing cells. It was widely expressed in the heart, blood vessels, kidney, muscle and other tissues, and participates in regulating lipid and carbohydrate and smooth muscle proliferation, migration and apoptosis, inflammation, atherosclerosis, and other pathologic processes [6]. Recent studies reported that PPARγ can regulate the blood pressure through renin–angiotensin–aldosterone system. Several studies have assessed the relationship between PPARγ gene polymorphism and primary hypertension [7–10]. These reports were not consistent because of differences in study populations, sample sizes, and so on. Yang et al. [11] had conducted a meta-analysis to quantitatively evaluate the relationship between the PPARγ2Pro12Ala (rs19813) polymorphism and hypertension. However, several important studies were not included in the analyses [10,12–18]. In the present study, we updated this evidence and gave more significant results.

## Materials and methods

This meta-analysis was in accordance with the Preferred Reporting Items for Systematic Reviews and Meta-Analyses (PRISMA) and Cochrane Handbook for Systematic Reviews.

### Literature search

We performed comprehensive computer-based searches in the PubMed, Web of Science, Embase, Google Scholar, the Cochrane Library, Wanfang database, China National Knowledge Infrastructure, and China Biological Medicine Database from the inception to 28 December 2018. All studies that reported the association between the PPARγ2Pro12Ala polymorphisms and hypertension were identified. The following keywords were used for searching: peroxisome proliferator-activated receptor-γ, PPAR-γ, Pro12Ala, genotype, polymorphism, mutation, single nucleotide polymorphism (SNP,) variant, hypertension. The search language was restricted to Chinese and English. We also reviewed the references lists of reviews and relevant studies for the potential studies as possible as we can.

### Criteria for inclusion and exclusion

The included studies have to meet the following criteria: (i) study is from a case–control or cohort study design about relationship between PPAR-γ2Pro12Ala polymorphism and the risk of hypertension; (ii) the diagnostic of hypertension is clear: systolic blood pressure ≥ 140 mmHg or diastolic blood pressure ≥ 90 mmHg without antihypertension medications or antihypertension medications were used [7]; (iii) study provides enough genotypes data of case group and control group for analysis; and (iv) the score is not less than 5 points according to the Newcastle–Ottawa Scale. We excluded the following studies: reviews, comments, letters, studies without enough data, quality score < 5. The latest data were used for duplicate data.

### Data extraction

Two authors [M.Z. and Q.W.] independently extracted the data. Disagreement was resolved by consensus. If these two authors could not reach a consensus, the result was reviewed by a third author [L.F.]. The extracted data consisted of the follow items: the first author’s name, publication year, country, methods of genotype, sample size of cases and controls, the gene frequency (PP, PA, AA) of case group and control group, Hardy–Weinberg equilibrium (HWE) test results, and scores of quality assessment.

### Quality assessment

To determine the methodological quality of each study, we used the Newcastle–Ottawa Scale, which uses a ‘star’ rating system to judge the quality of observational studies [19]. The NOS ranges from zero (worst) up to nine stars (best). Studies with a score equal to or higher than seven were of high quality. Two investigators (J.Z. and L.L.) independently assessed the quality of included studies, and the result was reviewed by a third investigator (L.F.). Disagreement was resolved by discussion.

### Statistical analysis

We assessed the association between PPAR gene Pro12Ala polymorphisms and risk of hypertension using the following gene model: dominant (AA/PA vs. PP), recessive (AA vs. PA/PP), additive (AA vs. PP), and allele gene model (A vs. P). The odds ratios (ORs) with 95% confidence intervals (CIs) were calculated to assess the relationship between gene models and risk of hypertension. Heterogeneity was assessed using the Chi-square test (significance level of *P*<0.1) and the *I^2^* test (greater than 50% as an evidence of significant inconsistency). Pooled effect sizes were determined using a fixed-effects model (the Mantel–Haenszel method) when heterogeneity was negligible (*I^2^* < 50%) or a random-effects model (the DerSimonian and Kacker method) when significant heterogeneity was present (*I^2^* ≥ 50%) [20]. HWE was assessed by the χ^2^ test in the controls. Subgroup analysis was also performed amongst different populations (Caucasian vs. Asian). We performed the sensitivity analysis by omitting one study each time. To study the relevance of such publication bias, funnel plots were constructed by plotting the trial results against their precision. Begg’s and Egger’s tests were also used to assess the publication bias [21]. A *P*-value less than 0.05 was considered statistically significant throughout the analyses.

### Ethical approval

This meta-analysis belonged to secondary analysis based on the studies published previously, the patients’ informed consent; and the ethical approval was not necessary.

## Results

### Study selection and general characteristics

**Figure 1:**
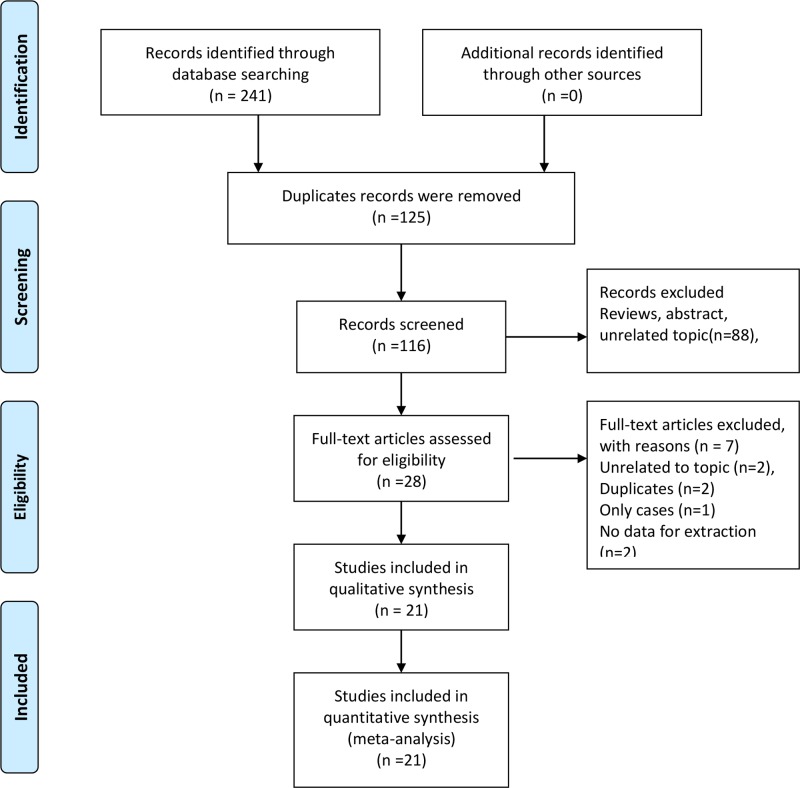
Flow chart of study selection

The flow of study selection was presented in Figure 1. Our initial search yielded 241 citations. Of these, 125 duplicate records were removed. One hundred and sixteen records were further screened via reviewing title and abstract. Eighty-eight studies were excluded because of results of interest not reported or other reasons. The remaining 28 studies were full-text reviewed, and 7 studies were excluded due to unrelated topics, duplicates, cases and no data for extraction. Finally, 21 studies met the inclusion criteria and were included in the meta-analysis [7–10,12–18,22–31].

The general characteristics of the studies included in the meta-analysis are shown in Table 1. Twenty-one studies consisted of 5503 cases and 7862 controls. These studies were published from 2003 to 2018. Fourteen studies were from China, two from Japan, and one each from Sweden, Spain, German, Poland, and Katar. The sample size of case ranged from 70 to 816, and control was from 112 to 1308. Most of studies used the PCR-restriction fragment length polymorphism (PCR-RFLP) to detect the genotype of PPARγ2Pro12Ala. The control selection of three studies did not conform to the HWE.

**Table 1:** General characteristics of studies included in the meta-analysis

Author	Year	Country	Methods of genotype	Sample size		Gene frequency (Case)			Gene frequency (Control)			HWE	Score of quality
				Case	Control	PP	PA	AA	PP	PA	AA		
Ostgren [22]	2003	Sweden	PCR-RFLP	194	190	147	43	4	136	51	3	0.468	8
Rodriguez-Esparragon [23]	2003	Spain	PCR	229	212	206	22	1	174	36	2	0.928	7
Horiki [7]	2004	Japan	PCR-RFLP	205	300	193	12	0	276	24	0	0.471	7
Shen [24]	2004	China	PCR-RFLP	125	112	113	11	1	103	9	0	0.658	7
Shen [12]	2004	China	PCR-RFLP	70	220	66	3	1	206	13	1	0.128	6
Zhang [25]	2005	China	PCR-RFLP	132	157	128	4	0	148	9	0	0.712	7
Gouni-Berthold [8]	2005	German	PCR-RFLP	255	140	190	57	8	104	32	4	0.430	7
Pan [26]	2007	China	PCR-RFLP	177	119	154	23	0	101	18	0	0.372	7
Hui [27]	2007	Jpan	TaqMan PCR	261	271	215	16	0	261	10	0	0.757	7
Lu [28]	2008	China	PCR-RFLP	478	361	446	31	1	312	48	1	0.550	7
Ruixing [13]	2008	China	PCR-RFLP	446	1213	418	23	5	1247	64	12	0.000	7
Gao [14]	2010	China	PCR-RFLP	345	137	337	7	1	131	2	4	0.000	7
Zhang [15]	2011	China	PCR-SSCP*	280	410	264	16	0	392	18	0	0.650	7
Dong [9]	2012	China	PCR-RFLP	124	178	122	2	0	177	1	0	0.970	7
Lian [16]	2012	China	TaqMan PCR	272	548	166	90	16	293	205	50	0.108	7
Bener [10]	2013	Katar	PCR	220	1308	185	28	7	1175	122	11	0.000	8
Gu [29]	2013	China	TaqMan PCR	269	551	166	85	18	293	210	48	0.262	7
Chen [30]	2014	China	MALDI-TOF-MS	145	165	110	33	2	105	53	7	0.924	6
Wang [31]	2015	China	PCR-RFLP	816	824	536	244	36	426	318	80	0.071	7
Grygiel-Gorniak [17]	2015	Poland	TaqMan PCR	151	120	101	44	6	84	32	4	0.661	7
Zhang [18]	2018	China	PCR-RFLP	309	290	262	45	2	259	31	0	0.336	8

*PCR-SSCP, Polymerase chain reaction single-strand conformation polymorphism.

### Assessment of quality

The score of each domain was presented in the Supplementary Material S1. The mean score of included studies was 7.04. The overall quality of studies was high. The main issues were that some studies selected a control population from a hospitalized one. Either cases and controls were not matched in the design and/or confounders were not adjusted for in the analysis. Besides, some studies did not report the non-response rate.

### Pooling results

**Figure 2:**
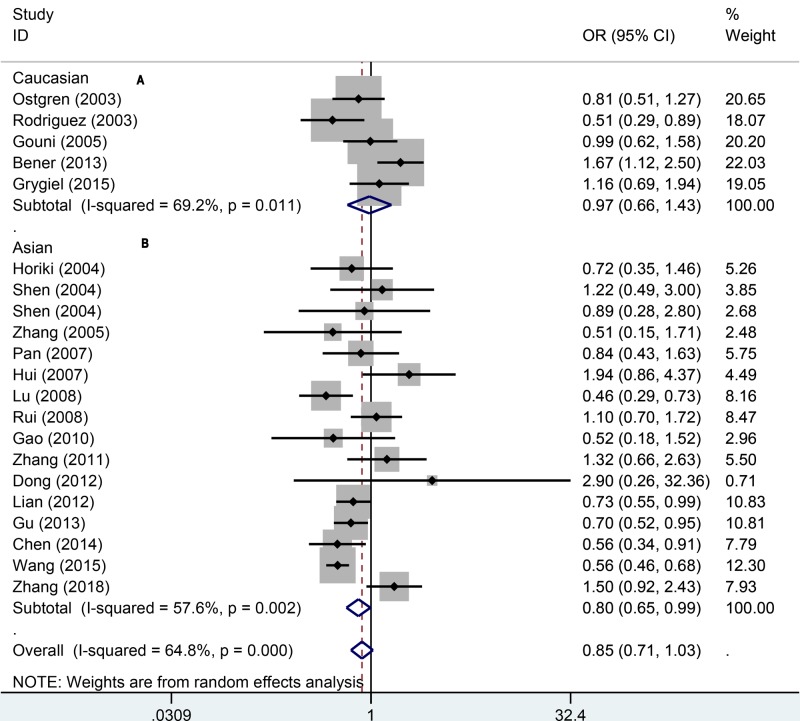
Forest plot for dominant effect model of association between PPAR gene Pro12Ala polymorphisms and risk of hypertension (A: Caucasian, B: Asian)

To quantitatively assess the association between PPAR-γ1Pro12Ala and hypertension, we first pooled 21 studies in the domain model (AA/AP vs. PP). The results indicated there was significant heterogeneity (*P*=0.000; *I^2^* = 64.8%). We used the random-effect model to pool the data, which suggested that the PPARγ1Pro12Ala polymorphism was not associated with hypertension (OR = 0.85, 95% CI: 0.71–1.03, *P*=0.108). To explore the population differences, we performed the subgroup analyses amongst different populations (Figure 2). In Caucasians, we pooled the data using the fixed-effect model because of moderate heterogeneity (*P*=0.011; *I^2^* = 69.2%). We found that the PPARγ1Pro12Ala polymorphism was also not related to the risk of hypertension. In Asians, however, the results from random-effect model (*P*=0.002; *I^2^* = 57.6%) indicated significant relationship between PPAR-γ1Pro12Ala and hypertension (OR = 0.80, 95% CI: 0.65–0.99).

**Figure 3:**
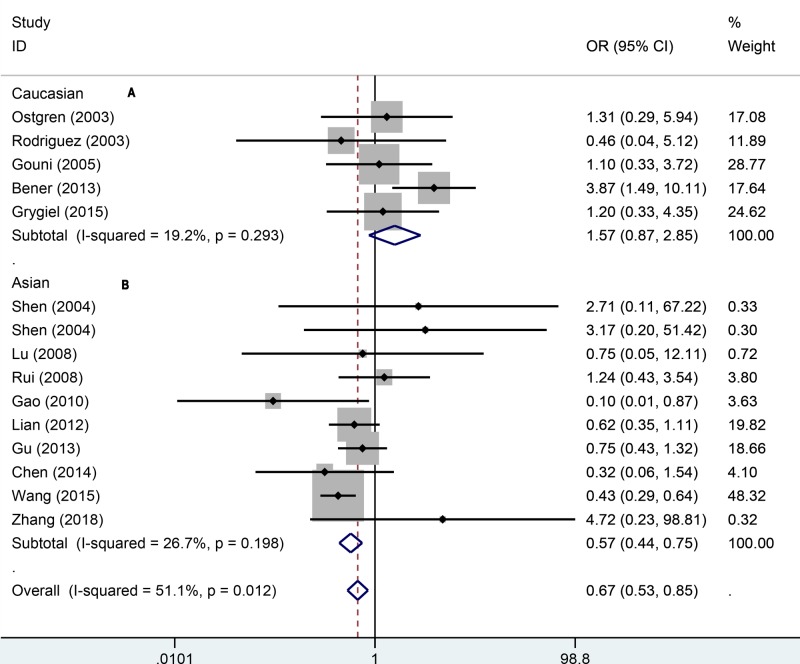
Forest plot for recessive effect model of association between PPARγ-2 gene Pro12Ala polymorphisms and risk of hypertension (A: Caucasian, B: Asian)

**Figure 4:**
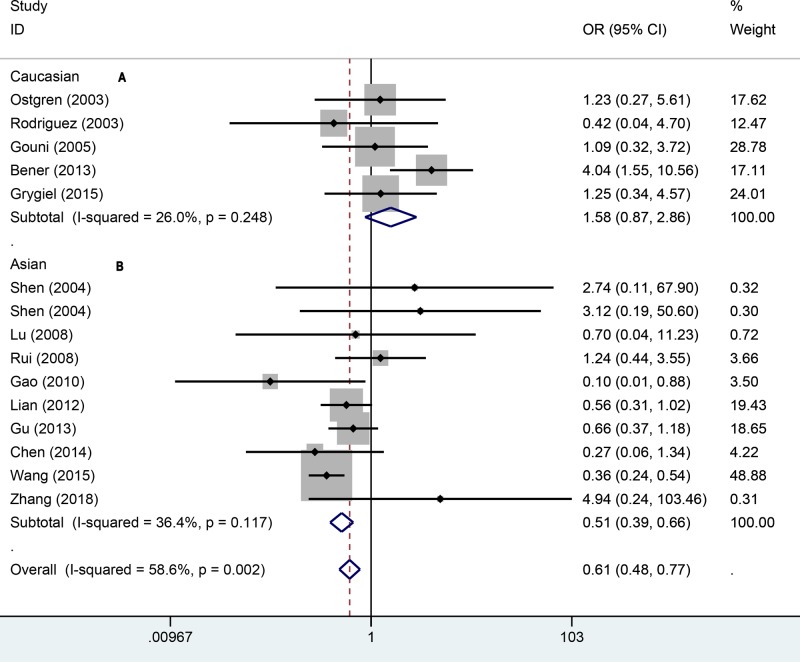
Forest plot for additive effect model of association between PPARγ-2 gene Pro12Ala polymorphisms and risk of hypertension (A: Caucasian, B: Asian)

**Figure 5:**
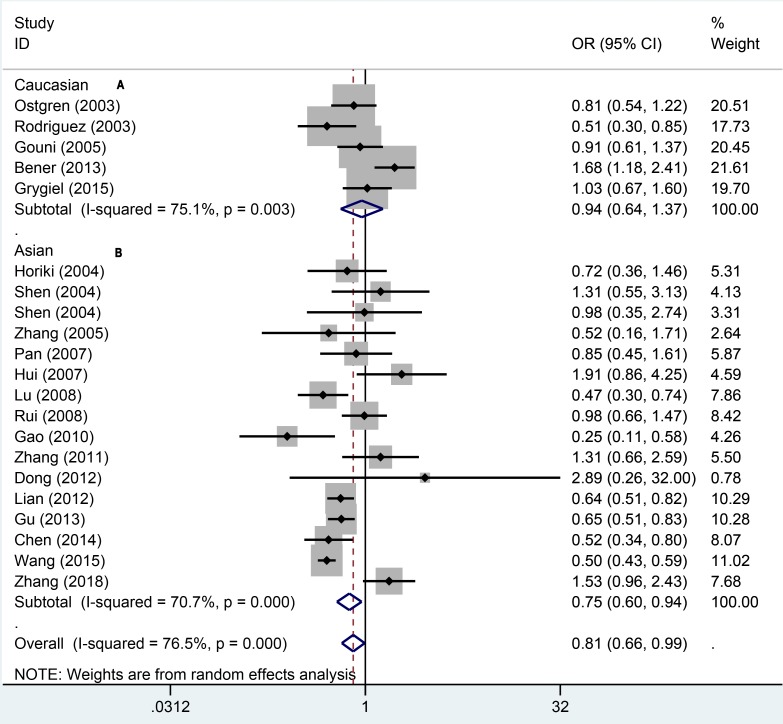
Forest plot for allele gene model of association between PPARγ-2 gene Pro12Ala polymorphisms and risk of hypertension (A: Caucasian, B: Asian)

To explore how the PPARγ1Pro12Ala polymorphism affects the hypertension, we also used other genetic models to assess the relationship. In the recessive model (Figure 3), a significant relationship was found via random-effect model (OR = 0.67, 95% CI: 0.53–0.85). The same result of fixed-effect model was observed in Asians (OR = 0.57, 95% CI: 0.44–0.75). There was no significance for Caucasians (OR = 1.57, 95% CI: 0.87–2.85). In the additive (Figure 4), the overall pooling data indicated significant results (OR = 0.61, 95% CI: 0.48–0.77). The AA genotype was also associated with hypertension risk amongst Asians (OR = 0.51, 95% CI: 0.39–0.66) under the fixed-effect model. For the Caucasians, the additive model suggested no significant relationship between AA genotype and hypertension (OR = 1.58, 95% CI: 0.87–2.56). In the allele genetic model (Figure 5), the overall pooling results suggested significant relationship (OR = 0.81, 95% CI: 0.66–0.99) and so did Asians (OR = 0.75, 95% CI: 0.60–0.94). Just like previous results, there was still no significance between PPAR-γ1Pro12Ala polymorphism and hypertension (OR = 0.94, 95% CI: 0.64–1.73). More details of pooling results are presented in the Table 2.

**Table 2:** Summary of different comparative results

Category	Genetic model		OR (95% CI)	Z	*P*-value	*I^2^* (%)	*P_heterogeneity_*	Effect model
Overall	Dominant	AA/PA vs. PP	0.85 (0.71–1.03)	1.61	0.108	64.8	0.000	Random
	Recessive	AA vs. PA/PP	0.67 (0.53–0.85)	0.65	0.518	51.1	0.012	Random
	Additive	AA vs. PP	0.61 (0.48–0.77)	4.06	0.000	58.6	0.002	Random
	Allele	A vs. P	0.81 (0.66–0.99)	2.08	0.038	76.5	0.000	Random
Asian	Dominant	AA/PA vs. PP	0.80 (0.65–0.99)	2.05	0.040	57.6	0.002	Random
	Recessive	AA vs. PA/PP	0.57 (0.44–0.75)	2.43	0.015	26.7	0.198	Fixed
	Additive	AA vs. PP	0.51 (0.39–0.66)	5.00	0.000	36.4	0.117	Fixed
	Allele	A vs. P	0.75 (0.60–0.94)	2.55	0.011	70.7	0.000	Random
Caucasian	Dominant	AA/PA vs. PP	0.97 (0.66–1.43)	0.14	0.887	69.2	0.011	Random
	Recessive	AA vs. PA/PP	1.57 (0.87–2.85)	1.49	0.136	17.4	0.304	Fixed
	Additive	AA vs. PP	1.58 (0.87–2.86)	1.50	0.134	26.0	0.248	Fixed
	Allele	A vs. P	0.94 (0.64–1.37)	0.33	0.743	75.1	0.003	Random

### Sensitivity analysis and publication bias

**Figure 6:**
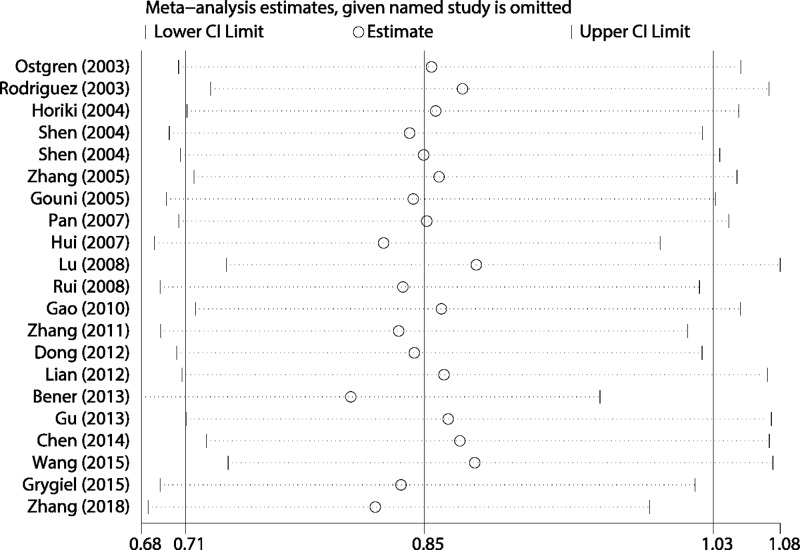
Sensitivity analysis for the overall pooled results based on dominant effect model

**Figure 7:**
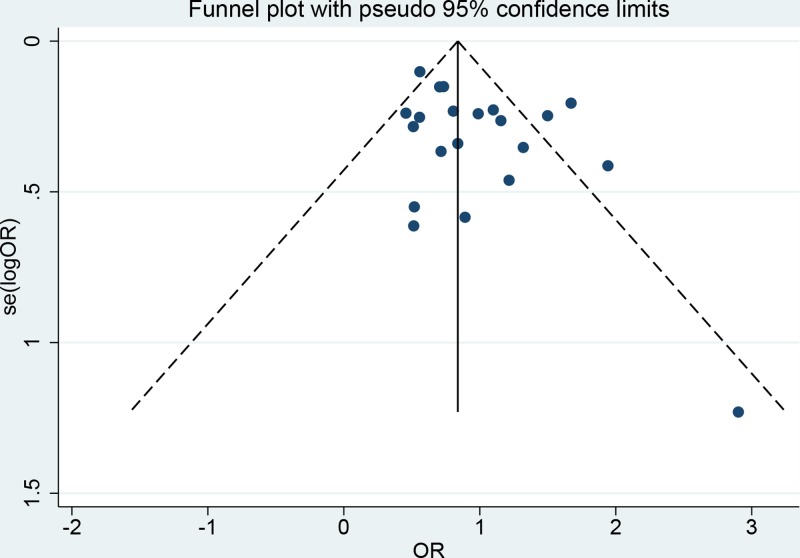
Funnel plot for publication bias

The sensitivity analysis result is presented in Figure 6. We performed the sensitivity analysis in the dominant because this model included all studies’ data. As we can see in the figure, the estimated results keep stable when given named study is omitted. The funnel plot indicated slight asymmetry, which means potential publication bias may exist (Figure 7). We also quantitatively assessed the publication bias using the Egger’s and Begg’s tests. The results suggested no potential publication bias (Z = 1.06, *P*=0.291; t = 1.98, *P*=0.062).

## Discussion

In the present study, our results based on larger sample sizes and more study number indicated that (i) PPAR-γ1Pro12Ala gene polymorphism was associated with the risk of hypertension. The AA genotype and A allele could decrease the risk of hypertension in the overall population compared with PP genotype and P allele, respectively. (ii) The population difference was source of heterogeneity within studies. Furthermore, (iii) the subgroup analysis suggested that the PPARγ1Pro12Ala gene polymorphism affects the risk of hypertension amongst Asians. However, no significant relationships were found amongst Caucasians. Our results provided more strong evidence support for the confirmation of relationship between PPARγ1Pro12Ala polymorphism and the risk of primary hypertension. Previous studies also assessed the relationship between common polymorphism (rs1801282) in the PPAR-γ2 gene and hypertension susceptibility [32,33]. However, this study only consisted of eight studies, and one of these studies was not in accordance with HWE. Several studies also included controls with diabetes mellitus and other diseases. The sensitivity analysis was not performed for this issue, which made the pooled results unstable. After that, Yang et al. [11] performed another investigation about this topic. They also just added five studies based on previous studies. The present study consisted of 21 studies. Although Yang et al. [11] also conducted subgroup analyses, the subgroup analyses were performed between China and Japan. Of course, they drew no significant difference between two countries. The present study performed subgroup between two populations (Asian and Caucasian) because the genetic background has more profound effects on hypertension susceptibility [30]. The pooled results confirmed our assumption. The PPARγ1Pro12Ala gene polymorphism tend to be associated with hypertension in Asians not Caucasians.

Our results found that the A allele appears to have a protective effect against hypertension. This result is similar to previous several studies. Lu et al. [28] found that Pro12Ala polymorphism of the PPAR-γ2 gene is associated with hypertension and triglycerides levels in Chinese nonagenarians/centenarians. The A allele frequency was significantly lower in the hypertension than that in the control group (3.45 vs. 6.92%) [28]. Bener et al. [10] investigated the association of the Pro12Ala polymorphism of the human PPARγ 2 (PPAR-γ2) gene with hypertension in a highly consanguineous aboriginal Qatari population. This study also revealed an association between the PPAR-γ2Ala allele and hypertension in Qatar’s population [10]. Paralleled with Lu et al.’s [28] study, Wang et al. [31] conducted a case–control study on PPARγ polymorphism and essential hypertension in Chinese Han. They also found that the A allele of PPAR-γ was associated with lower risk of essential hypertension [31]. On the contrary, Li et al. [18] recently investigated the relationship between the polymorphism of peroxisome proliferators-activated receptor2 gene Pro12Ala and essential hypertension in Hui and Han nationality in Ningxia Province. This study reported that there was no correlation between Pro12Ala polymorphism of PPAR-γ 2 gene and essential hypertension Hui and Han population in Ningxia. Furthermore, this gene polymorphism had no racial difference between Hui and Han populations in Ningxia [18]. These results indicated the effect of Pro12Ala is also different even under the same genetic background. The underlying mechanism for the protective effect of the Ala allele remains speculative. The reasons could be explained by the following several mechanisms. First, PPAR-γ can inhibit the growth of vascular cells. Most cells from blood vessel can express PPAR-γ that induces apoptosis of vascular smooth muscle cells (VSMC) and improves the vascular structure. The infusion of Ang II can increase arterial blood vessels middle/space ratio, and this situation can be relieved through the usage of rosiglitazone and pioglitazone. PPAR-γ also can degrade AT1 receptor expression and inhibit DNA synthesis stimulated by Ang II by adjusting the cyclin-dependent kinase inhibitor. Rosiglitazone can induce VSMC apoptosis, which may be related to this effect [34]. Second, PPAR can affect the renin–angiotensin system (RASS) via the following several pathways: inhibiting the expression of angiotensinogen, inhibiting Ang II activity, and degrading the angiotensin receptor I expression in the VSMCs. *In vitro*, high-salt diet can make the SHR blood pressure significantly increased (185 + 6) mmHg vs. (128 ± 5) mmHg, and this process can be blocked by Rosiglitazone block (126 + 4 mmHg). Ang II synthetic process and glomerular mesangial cell proliferation was also inhibited, which indicated that the role in antihypertensive and protecting renal was associated with Ang II. Diep et al. [35] found that Rosiglitazone can reverse the elevation blood pressure effect caused by Ang II. Third, PPAR-γ can regulate the blood pressure by regulating vascular vasodilators and vasoconstrictors secretion. PPAR-γ can stimulate endothelial cells to secrete type-C natriuretic peptide (CNP). C peptide is one of diuresis sodium peptide family members, produced by endothelial cells, is a kind of new in the original diastolic peptide. Previous study found that CNP gene transfection mediated by adenovirus can significantly inhibit the proliferation of VSMCs [36]. During the process of coronary artery atherosclerosis formation, the CNP expressed by endothelial cell also gradually reduced. However, Triglidone and Pioglitazone can stimulate the secretion of CNP by endothelial cells. The present findings were from the effect of PPAR-γ agonist on blood pressure [37]. The direct function mechanism of PPAR in regulating blood pressure needs further research.

The present study has several strengths. Compared with previous studies, our study consisted of a larger sample size (5503 cases and 7862 controls), which improved the statistical power and provided more accurate results. We also conducted subgroup analysis that were not reported in previous study. Besides, the present study followed the PRISMA and Cochrane Handbook for Systematic Reviews. Several study limitations should be addressed. First, the present pooled results were from unadjusted data, and some potential confounding factors may affect the results. We confirm that the population difference was one of the potential confounding factors. Second, we can see publication bias may exist though the Egger’s and Begg’s tests gave no significant results. Maybe it was related to search language because we only performed search in English and Chinese. Third, the study number and sample size in Caucasians was very limited. Studies with larger sample sizes are needed. Finally, future study should take the gene–environment effects into consideration.

In conclusion, the PPAR-γ2 Pro12Ala polymorphism could affect the risk of primary hypertension amongst Asians. The A allele gene was a protective genotype in primary hypertension. The PPAR-γ1Pro12Ala polymorphism was not associated with hypertension amongst Caucasians. Study with more sample size is needed, especially for Caucasians. The future research should pay attention on the molecular mechanisms behind this.

## Supporting information

**Supplementary material 1:** Methodological quality assessment (risk of bias) of included studies by Newcastle-Ottawa Scales

## Abbreviations

Ang II: Angiotensin II
AT I: Angiotensin type 1 receptor
CI: Confidence interval
CNP: Type-C natriuretic peptide
HWE: Hardy–Weinberg equilibrium
NOS: Nitric oxide synthase
OR: Odds ratio
PPAR: Peroxisome proliferator-activated receptor
PRISMA: Preferred Reporting Items for Systematic Reviews and Meta-analyses
SHR: Spontaneously hypertensive rat
VSMC: Vascular smooth muscle cell
