# Supplementary material for: Association between peroxisome proliferator-activated receptor γ-2 gene Pro12Ala polymorphisms and risk of hypertension: an updated meta-analysis
Source: Biosci Rep. 2019 Feb 27;39(2):BSR20190022. doi: 10.1042/BSR20190022 (PMC6393226; doi:10.1042/BSR20190022)
Supplement: Supplementary file 1 [file bsr-39-bsr20190022_Supp1.pdf]

Supplementary material 1 Methodological quality assessment (risk of bias) of included studies by Newcastle-Ottawa Scales

Study	Selection				Comparability	Exposure		
	Selection of Cases	Representativeness	Selection of Controls	Definition of Controls		Ascertainment of Exposure	Same method of ascertainment	Non-Response Rate
Ostgren	*	*	*	*	*	*	*	*
Rodriguez	*	*	*	*	*	*	*	-
Horiki	*	*	*	*	*	*	*	-
Shen	*	*	-	*	*	*	*	-
Shen	-	*	*	-	*	*	*	*
Zhang	*	*	*	*	*	*	*	-
Gouni	*	*	-	*	*	*	*	*
Pan	*	*	*	*	*	*	*	-
Hui	*	*	*	*	*	*	*	-
Lu	*	*	-	*	*	*	*	*
Rui	*	*	-	*	*	*	*	-
Gao	*	*	-	*	*	*	*	*
Zhang	*	*	*	*	*	*	*	-
Dong	*	*	-	*	*	*	*	*
Lian	*	*	*	*	*	*	*	-
Bener	*	*	-	*	**	*	*	*
Gu	*	*	*	*	*	*	*	-
Chen	*	*	-	*	*	*	*	-
Wang	*	*	*	*	*	*	*	-
Grygiel	*	*	*	*	*	*	*	-
Zhang	*	*	*	*	*	*	*	*
